# Live Imaging of Calciprotein Particle Clearance and Receptor Mediated Uptake: Role of Calciprotein Monomers

**DOI:** 10.3389/fcell.2021.633925

**Published:** 2021-04-29

**Authors:** Sina Koeppert, Ahmed Ghallab, Sarah Peglow, Camilla Franziska Winkler, Steffen Graeber, Andrea Büscher, Jan Georg Hengstler, Willi Jahnen-Dechent

**Affiliations:** ^1^Helmholtz-Institute for Biomedical Engineering, RWTH Aachen University, Aachen, Germany; ^2^Leibniz Research Centre for Working Environment and Human Factors, Dortmund, Germany; ^3^Department of Forensic Medicine and Toxicology, Faculty of Veterinary Medicine, South Valley University, Qena, Egypt

**Keywords:** calciprotein monomer, calcification, fetuin-A, plasma protein, mineral metabolism, calciprotein particle

## Abstract

**Background:**

The liver-derived plasma protein fetuin A is a systemic inhibitor of ectopic calcification. Fetuin-A stabilizes calcium phosphate mineral initially as ion clusters to form calciprotein monomers (CPM), and then as larger multimeric consolidations containing amorphous calcium phosphate (primary CPP, CPP 1) or more crystalline phases (secondary CPP, CPP 2). CPM and CPP mediate excess mineral stabilization, transport and clearance from circulation.

**Methods:**

We injected i.v. synthetic fluorescent CPM and studied their clearance by live two-photon microscopy. We analyzed organ sections by fluorescence microscopy to assess CPM distribution. We studied cellular clearance and cytotoxicity by flow cytometry and live/dead staining, respectively, in cultured macrophages, liver sinusoidal endothelial cells (LSEC), and human proximal tubule epithelial HK-2 cells. Inflammasome activation was scored in macrophages. Fetuin A monomer and CPM charge were analyzed by ion exchange chromatography.

**Results:**

Live mice cleared CPP in the liver as published previously. In contrast, CPM were filtered by kidney glomeruli into the Bowman space and the proximal tubules, suggesting tubular excretion of CPM-bound calcium phosphate and reabsorption of fetuin A. Fetuin-A monomer clearance was negligible in liver and low in kidney. Anion exchange chromatography revealed that fetuin A monomer was negatively charged, whereas CPM appeared neutral, suggesting electrochemical selectivity of CPM versus fetuin A. CPM were non-toxic in any of the investigated cell types, whereas CPP 1 were cytotoxic. Unlike CPP, CPM also did not activate the inflammasome.

**Conclusions:**

Fetuin-A prevents calcium phosphate precipitation by forming CPM, which transform into CPP. Unlike CPP, CPM do not trigger inflammation. CPM are readily cleared in the kidneys, suggesting CPM as a physiological transporter of excess calcium and phosphate. Upon prolonged circulation, e.g., in chronic kidney disease, CPM will coalesce and form CPP, which cannot be cleared by the kidney, but will be endocytosed by liver sinusoidal endothelial cells and macrophages. Large amounts of CPP trigger inflammation. Chronic CPM and CPP clearance deficiency thus cause calcification by CPP deposition in blood vessels and soft tissues, as well as inflammation.

## Introduction

The formation of stable protein mineral complexes, named calciprotein particles (CPP) is considered as potent mechanism to prevent soft tissue calcification (Heiss et al., 2008). CPP consist of calcium, phosphate, fetuin-A, and further plasma proteins. In the first maturation state, CPP form as colloidal nanoparticles called primary CPP or CPP-1. Time-, pH-, temperature-, and mineral saturation-dependently, CPP-1 undergo an Ostwald ripening process associated with structural and compositional rearrangements resulting in crystalloid secondary CPP or CPP-2 (Wald et al., 2011). CPP-2 are ellipsoid shaped with a crystalline core surrounded by a protein layer (Heiss et al., 2003) (Supplementary Figure 1). With particle ripening, the protein composition of CPP will change (Smith et al., 2018). Fetuin-A, albumin, and the plasma proteins apolipoprotein A1, prothrombin, and complement C3 were consistently identified across multiple studies (Wu et al., 2013). CPP-1 have a hydrodynamic diameter of approximately 50–100 nm, and CPP-2 of 100–300 nm (Wald et al., 2011).

Small angle neutron scattering analysis of CPP topology, composition, and morphogenesis revealed that fetuin-A stabilized the fluid phase in conditions of calcium and phosphate supersaturation, in that 5–10% of the fetuin-A protein and about one half of mineral ions present in the initial mixture ultimately comprised CPP. The second half of mineral ions formed small calcium phosphate clusters bound to fetuin-A monomers. These particles were named calciprotein monomers CPM (Heiss et al., 2010). Recent research demonstrated that protein-sized CPM may actually be the predominant circulating form of protein-mineral complexes in the body (Miura et al., 2018; Smith et al., 2018). These are much smaller at 9–10 nm diameter and may thus be cleared differently from both CPP-1 and CPP-2. Clearance studies of synthetic nanoparticles showed that the preferred clearance mechanisms strongly depends on size, charge, geometry, and protein absorption (Chithrani et al., 2006; Behzadi et al., 2017). Nanoparticles are cleared through three principal mechanism, the mononuclear phagocyte system (MPS), renal filtration, and the hepatobiliary elimination (Zhang et al., 2016). The MPS comprises professional phagocytic mononuclear cells originating from the bone marrow, which mobilize into the circulation and ultimately reside in tissues as macrophages (Lasser, 1983). Professional phagocytes include macrophage related bone osteoclasts, brain microglia, liver Kupffer cells, dendritic cells in skin and lymph nodes, as well as lung, spleen, bone marrow, gut, and peritoneal fluids macrophages (Lasser, 1983; Gordon et al., 1988). MPS macrophages clear stiff particles of 200 nm diameter and larger, including bacteria and rigid nanoparticles, as well as soft particles of up to 200 nm diameter, including cell remnants and apoptotic cell vesicles, platelet fragments, aged red cells, and protein complexes. To leave circulation into renal excretion, nanoparticles must be small enough to pass the fenestrated endothelium, the glomerular basement membrane and the epithelial filtration slits between podocyte extensions, and several layers of charge-repulsive glomerular capillary wall. Ultrasmall engineered nanoparticles of less than 5 nm diameter including quantum dots (Fridén et al., 2011) or ultrasmall gold nanoparticles (Hausmann et al., 2010) passed the renal filtration barrier into the urine. Due to negative charge of the glomerular capillary wall, surface charge of nanoparticles was equally important like particle size and shape (Jinbin et al., 2013). The clearance studies with synthetic nanoparticles corroborated classical ultrafiltration and surface charge rejection theory of renal filtration, which basically states that in the physiological state uncharged particles of up to 3.5 nm diameter freely pass the endothelial barrier of the kidney glomerulus into primary urine, while larger and negatively charged particles do not (Hausmann et al., 2010; Fridén et al., 2011; Tenten et al., 2013).

Previously we showed that liver sinusoidal endothelial cells (Koeppert et al., 2018) and liver Kupffer cells cleared CPP-1 and CPP-2, respectively (Herrmann et al., 2012). A clearance mechanism for CPM was never studied due to the lack of stability of CPM preparations for use in animal and cell-based studies. CPM are small enough to pass the renal filtration barrier, but the overall negative charge of fetuin-A (Heiss et al., 2003), the major protein component of CPM should prevent by charge rejection renal clearance. Nevertheless, we observed renal clearance of CPM in live mice and in cell culture. We propose a differential clearance mechanism of fetuin-A monomer vs. CPM based on charge neutralization.

## Materials and Methods

### Cell Culture

Immortalized wildtype macrophages and immortalized macrophages expressing inflammasome adaptor protein apoptosis-associated speck like protein containing a caspase recruitment domain fused to greed fluorescent protein (ASC-GFP, a kind gift from Eicke Latz, MD Ph.D., Institute of Innate Immunity, Bonn, Germany) were cultured in Roswell Park Memorial Institute (RPMI-1640, 21875034, Thermo Fisher Scientific GmbH, Dreieich, Germany) supplemented with 10% FCS, 100 U penicillin, and 100 μg streptomycin.

Immortalized liver sinusoidal endothelial cells (Applied Biological Materials Inc., Richmond, Canada, a kind gift from Dr. Marie-Luise Berres, Medical Clinic III, RWTH University Hospital Aachen, Germany), were cultured in Prigrow I Medium (Applied Biological Materials Inc., Richmond, Canada) supplemented with 5% FCS, 100 U penicillin, and 100 μg streptomycin and grown on collagen l-coated cell culture plates.

Immortalized human kidney 2 cells (HK-2, kindly provided by Prof. Peter Boor, Institute for Pathology, RWTH University Hospital Aachen, Germany) were grown in DMEM GlutaMAX (10566016, Thermo Fisher Scientific GmbH, Dreieich, Germany) supplemented with 5% FCS, 100 U penicillin, and 100 μg streptomycin.

### Animals

All animal experiments were conducted in agreement with the recommendation of the Federation for Laboratory Animal Science Associations (FELASA), and were approved by the animal welfare committee of the Landesamt für Natur-, Umwelt- und Verbraucherschutz (LANUV, 84-02.04.2013.A113 and 84.02.04.2015.A294). At least three male or female, adult C57Bl/6 mice each were injected in the clearance experiments. All mice were maintained in a temperature-controlled room on a 12-h day/night cycle. Food and water were given *ad libitum*.

### Fetuin-A Purification and Labeling

Bovine fetuin-A (F2379, Sigma-Aldrich, St. Louis, MO, United States) was purified by gel permeation chromatography as described previously (Heiss et al., 2003). Purified fetuin-A was routinely analyzed for LPS activity using the Endosafe ultrasensitive cartridge assay (Charles River Laboratories, Wilmington, DE, United States). LPS content was <0.1 EU/ml in all fetuin-A preparations and did not induce cytokine secretion in macrophages.

For visualization, purified fetuin-A was labeled with Alexa488 or Alexa546 NHS ester (A20000, A20104, Thermo Fisher Scientific GmbH, Dreieich, Germany) according to manufacturer’s protocol. Fetuin-A monomer was used to prepare CPP-1 and CPP-2 as well as CPM.

### Calciprotein Monomer Synthesis and Purification

Bovine fetuin-A-derived CPM were prepared in a solution containing 400 μl 2.5 mg/ml fetuin-A in 140 mM sodium chloride mixed with additional 100 μl 140 mM sodium chloride and 250 μl 24 mM phosphate buffer (pH 7.4). After thorough mixing 250 μl of 40 mM Ca chloride solution (pH 7.4) were added and mixed again. Final concentrations per 1 ml mixture were 1 mg bovine fetuin-A, 6 mM phosphate, and 10 mM Ca. CPM formation proceeded at 37°C for 10 min. This reaction will contain CPP-1, CPM and free fetuin-A monomer. The complete mixture was desalted using spin filtration devices with a 3,000 MW cutoff (Vivaspin 2, VS0251, Sartorius AG, Göttingen, Germany). CPP-1 were separated using spin filtration devices with a 300,000 MW cutoff (Vivaspin 2, VS0291, Sartorius AG, Goettingen, Germany). The flowthrough typically contains a mixture of about 10% CPM and about 90% free fetuin-A monomer, which could not be further separated. This CPM/fetuin-A monomer fraction from here on is called CPM for simplicity. Because the CPM preparation contained a large portion of free fetuin-A monomer, an additional fetuin-A monomer control was included in all assays. CPP were prepared from an identical protein-mineral mixture. CPP-1 and CPP-2 were harvested by centrifugation (20,000 × *g* for 15 min at 4°C) after 10 min and overnight incubation, respectively.

### Calcium- and Phosphate Measurement

Calcium content was quantified using the Randox Calcium Assay (Randox Laboratories, Krefeld, Germany) according to manufacturer’s protocol. Briefly, 5 μl of samples and standards (0–2.5 mM Ca) were added to 200 μl working solution (Randox Laboratories, Krefeld, Germany) and analyzed in duplicates in 96-well plates. Plates were measured in a plate reader with a wavelength of 570 nm.

Phosphate was quantified using a photometric UV-test (533-940, mti-diagnostics, Wiesbaden, Germany) according to manufacturers’ protocol. Shortly, 5 μl sample and standards (0–1.61 mM phosphate) were added to 240 μl working solution and analyzed in duplicates in 96-wells plates. Plates were measured at 340 nm.

### Live 2-Photon Microscopy Imaging

Functional intravital imaging of mouse organs and image analysis was performed as described (Reif et al., 2017; Koeppert et al., 2018; Ghallab et al., 2019) using a customized inverted microscope LSM MP7 (Zeiss, Jena, Germany) with an LD C-Apochromat 40 × 1.1 water immersion objective. Videos were recorded using a two-photon microscope and a video camera. Fluorescence intensity was recorded using a GaAsP detector. Briefly, mice expressing membrane-anchored tdTomato in all cells, were anesthetized by i.p. injection of ketamine (100 mg/kg bodyweight), xylazine (10 mg/kg bodyweight), acepromazine (1.7 mg/kg bodyweight), and buprenorphine (0.08 mg/kg bodyweight). Anesthesia was maintained throughout the observation period using an isoflurane inhaler. The animals received i.v. injections of Hoechst 33258 (5 mg/kg) to stain nuclei. Few seconds after starting image acquisition, Alexa488-tagged CPM were injected i.v. using a catheter inserted into the tail vein. Mice were injected with an equivalent of 100 μg CPM-associated fetuin-A (900 μg total protein including 800 μg free fetuin-A monomer), 100 and 900 μg free fetuin-A monomer in a maximum volume of 200 μl. Intravital 2-photon microscopy videos of the kidney were continuously recorded. Experiments were performed with at least three mice.

### *Ex vivo* Fluorescence Imaging

Mice were anesthetized with an i.p. injection of ketamine (100 mg/kg bodyweight), xylazine (10 mg/kg bodyweight) and received i.v. injections of a maximum volume of 200 μl. To block lysosomal degradation mice were injected i.v. with 0.03 mg/g bodyweight leupeptin 30 min before injection with protein-mineral complexes. Single boluses containing 100 μg Alexa488- or Alexa546-labeled fetuin-A monomer, or CPM, or CPP-1, or CPP-2 were injected. In the case of CPM, a total amount of up to 900 μg labeled fetuin-A containing ∼100 μg CPM-bound fetuin-A was injected. Mice injected with 900 μg labeled fetuin-A monomer for control purposes showed essentially the same results like mice injected with 100 μg fetuin-A monomer. Ten minutes post injection, mice were sacrificed by isoflurane overdosing and organs were collected for sectioning. Tissues were imbedded in Tissue-Tek O.C.T. compound (Sakura Finetek Germany GmbH, Staufen, Germany) and 5 μm cryosections were prepared. Sections were counterstained with DAPI, mounted with Immomount^®^ (Thermo Fisher Scientific, Dreieich, Germany) and analyzed by fluorescence microscopy.

### Endocytosis Assay

Cells were seeded in 0.5 ml cell culture medium at a density of 250,000 cells/ml in 24-well plates and kept overnight. The next day, the cells were incubated for 1 h with 100 μg Alexa488-labeled fetuin-A monomer, CPP-1, or CPP-2 or with a combination of CPP-1 and CPP-2. CPM were applied in amounts as described in Imaging. Cells were washed twice with PBS and observed under a fluorescence microscope. After cell detaching fluorescent fetuin-A monomer, CPM, and CPP uptake was measured by flow cytometry.

### Flow Cytometry

Following endocytosis assay, cell-associated fluorescence after fetuin-A monomer/CPM/CPP uptake was analyzed by flow cytometry using a FACSCalibur (BD Biosciences, San Jose, CA, United States) flow cytometer equipped with a 488 nm argon-ion laser. To examine the Alexa488-labeled fetuin-A monomer, CPM, or CPP, the green channel (FL-1H) was used. For each sample 10,000 cells were evaluated. Data were analyzed using FlowJo software.

### Live/Dead Cell Staining

Immortalized wildtype macrophages, LSEC and HK-2 cells were seeded at a density of 200,000 cells/ml in the cell culture media listed above (section “Cell Culture”) on 24-well plates and kept overnight. The following day, cells were treated with calcium content matched (2.5 mM) CPM, CPP-1, and CPP-2, and controls (cell culture medium, fetuin-A monomer, 2.5 mM calcium, 2.0 mM phosphate, calcium-phosphate) for 8 h. Afterward, cells were stained with a mixture of 0.5 μg/ml fluorescein diacetate (FDA, Thermo Fisher Scientific GmbH, Dreieich, Germany) and 0.05 μg/ml propidium iodide (PI, Thermo Fisher Scientific GmbH, Dreieich, Germany) in PBS for 30 s. Following staining, cells were washed twice with PBS and examined by fluorescence microscopy.

### Inflammasome Activation Assay

Immortalized macrophages expressing inflammasome adaptor protein apoptosis-associated speck like protein containing a caspase recruitment domain fused to green fluorescent protein (ASC-GFP) were used to visualize inflammasome activation. If the inflammasome is inactive ASC is localized in the cytoplasm and nucleus of the cell detectable as a weak diffuse fluorescent signal in the whole cell. Inflammasome activation results in the recruitment of the fluorescently tagged adaptor protein ASC leading in the formation of a single green-fluorescent speck per cell.

ASC-GFP macrophages were seeded at a density of 250,000 cells/ml in RPMI-1640 medium supplemented with 10% FCS on 24-well plates and kept overnight. The next day, cells were treated with either medium control, 10 μM nigericin (InvivoGen Europe, Toulouse, France), or with calcium content matched (2.5 mM) CPM, CPP-1 or CPP-2. After 2-24 h, speck formation was visualized using fluorescence microscope.

### Ion Exchange Chromatography

Fetuin-A monomer and CPM preparations were analyzed using ion exchange chromatography (IEX). Free bovine fetuin-A monomer or CPM preparations (both containing 900 μg total protein) dissolved in 1 ml 150 mM sodium chloride were loaded at 4 ml/min flow onto a ResourceQ anion exchange column (1 ml column volume) using an Äkta Pure chromatography instrument (GE Healthcare Europe GmbH, Freiburg, Germany). After rinsing with 10 ml running buffer (20 mM Tris pH 8.0), the column was eluted with 8 ml 0.5 M NaCl, followed by 10 ml 1 M NaCl solution. All chromatography buffers were filtered and degassed using 0.45 μm microfilters and a vacuum flask.

## Results

### Live Imaging of Glomerular Filtration and Proximal Tubule Re-absorption of Calciprotein Monomers and Fetuin-A

**FIGURE 1:**
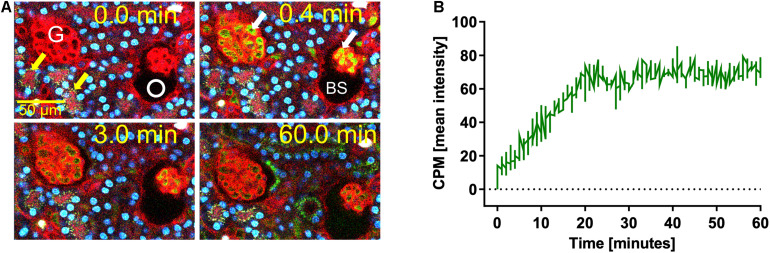
Renal CPM clearance by glomerular filtration. Mice expressing membrane-anchored tdTomato (red) in all cells received intravenous injections of CPM prepared with Alexa488-labeled bovine fetuin-A (green). Nuclei were visualized by Hoechst 33258 (blue). Videos were recorded using a two-photon microscope and a video camera. Fluorescence intensity was recorded using a GaAsP detector. **(A)** Glomerular CPM filtration at 0, 0.4, 3, and 60 min post injection. **(B)** Time-resolved CPM fluorescence in the Bowman space (BS) recorded within the white circle shown in panel **(A)** demonstrates CPM filtration reaching a plateau at 20 min. Yellow autofluorescence in distal tubules [yellow arrows in panel **(A)**] was already visible before CPM injection. Still pictures illustrating CPM glomerular filtration in panel **(A)** were taken from Supplementary Movie 1.

**FIGURE 2:**
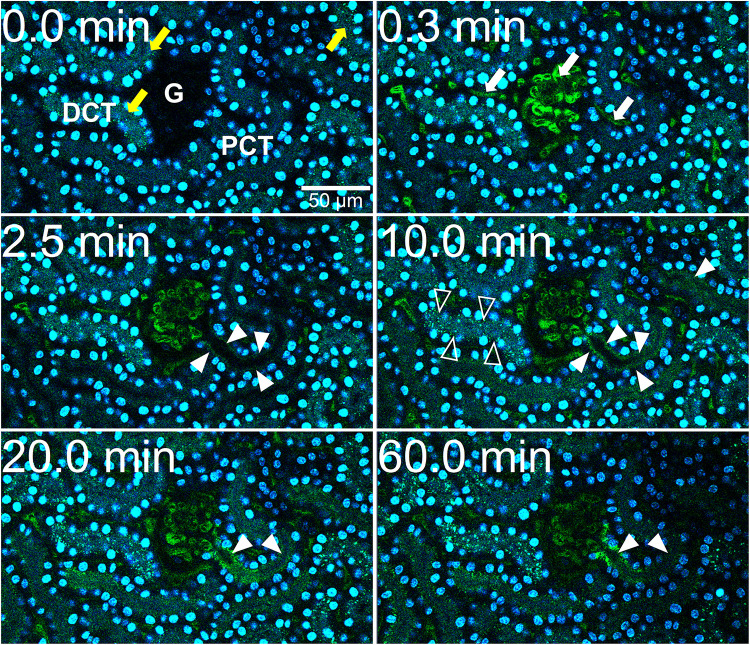
CPM-derived fetuin-A is reabsorbed in proximal renal tubules. Mice received intravenous injections of CPM prepared with Alexa488-labeled bovine fetuin-A (green). Nuclei were visualized by Hoechst 33258 (blue). Videos were recorded using a two-photon microscope and a video camera. Fluorescence intensity was recorded using a GaAsP detector. A renal filtration unit was continuously monitored up to 60 min. Representative micrographs demonstrating a typical sequence of fetuin-A tubular reabsorption. Yellow autofluorescence in distal tubules (yellow arrows, *t* = 0.0 min) was already visible before CPM injection. Within seconds after injection, green signal was detectable in glomerular and peritubular capillaries (white arrows, *t* = 0.3 min). At 2.5 min, proximal tubular (PCT) epithelial cells (filled white arrowheads) became green fluorescence positive showing a steadily increasing signal until 10 min, whereas distal tubular (DCT) cells showed no green fluorescent signal (white empty arrowheads), suggesting cellular uptake of green CPM-associated fetuin-A protein by proximal tubular epithelial cells from the tubule lumen. Fluorescence remained strong at the origin of the proximal tubuli where they connect to the glomeruli, while fluorescence continuously decreased in glomerular and peritubular capillaries until 60 min. Photographs illustrating proximal tubule re-uptake were taken from Supplementary Movie 2.

We previously demonstrated that liver sinusoidal endothelial cells, and spleen and liver macrophages (Kupffer cells) clear CPP-1 and CPP-2, respectively (Herrmann et al., 2012; Koeppert et al., 2018). Clearance of CPM was never analyzed, because CPM are inherently unstable and laborious to make. We prepared CPM by mixing Alexa488- (red) fetuin-A and a mineral precipitation mixture, and size-fractionated the preparation by ultra-filtration. We injected intravenously 100 μg of CPM-bound fetuin-A (900 μg total protein) in mice. On average, CPM preparations contain up to 90% mineral-free fetuin-A monomer, which cannot be size-separated. To assess the contribution of this fetuin-A monomer fraction to overall clearance, we also injected mice with Alexa488-labeled fetuin-A monomer alone. To study renal clearance in real time we injected live C57BL/6 mice expressing membrane-anchored tdTomato (red) in all cells, with 100 μg CPM prepared with Alexa488-tagged (green) fetuin-A (900 μg total protein) or with 900 μg free fetuin-A, and monitored kidney fluorescence using intravital 2-photon microscopy. Mice received in addition Hoechst 33258 dye (blue) to visualize nuclei. Imaged kidneys showed punctate autofluorescence in distal tubules (yellow arrows in Figures 1A, 2, *t* = 0.0 min). Autofluorescence was clearly distinguishable from the green CPM-associated Alexa488-tagged fetuin-A signal. Figure 1 shows a glomerular filtration unit continuously recorded from timepoint 0 immediately before CPM injection up to 60 min post CPM injection. Figure 1A and Supplementary Movie 1 show rapid filtration of CPM from glomerular capillaries (white arrows in Figure 1A, *t* = 0.4 min) into the Bowman space (BS) within seconds post injection. Figure 1B illustrates fluorescence quantitation (measured in the white circled area in Figure 1A) of the BS showing that glomerular CPM filtration began immediately after injection and plateaued at 20 min post injection. From 20 min post injection, the measured signal in the BS stayed constant until the end of the recording time at 60 min. Subsequently, the filtered green-fluorescent CPM appeared in the epithelial cells of the proximal tubules (filled white arrowheads in Figure 2, *t* = 2.5 min; Supplementary Movie 2, whereas distal tubular cells showed no green fluorescence (empty white arrowheads in Figure 2, *t* = 10 min). This finding indicated re-absorption of CPM-associated Alexa488-tagged fetuin-A by proximal tubular epithelial cells from tubule lumen. From 20 min post injection, the green-fluorescent signal decreased along the proximal tubules until 60 min post injection (white arrowheads in Figure 2). Notably, the fluorescent signal remained high at the glomerular junction of the proximal tubule, while the signal continuously declined in glomerular and peritubular capillaries until 60 min post injection (Figure 2 and Supplementary Movie 2), suggesting continuous glomerular filtration of CPM and tubular re-uptake of fetuin-A.

**FIGURE 3:**
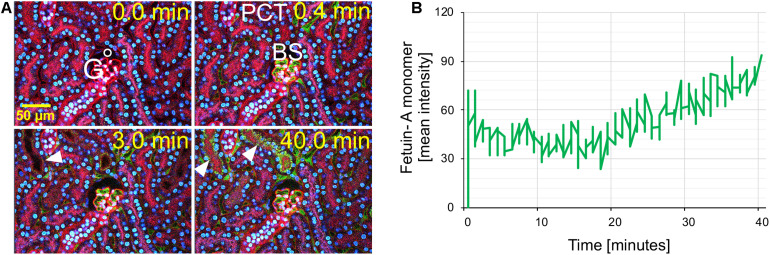
Renal fetuin-A monomer clearance by glomerular filtration. Mice expressing membrane-anchored tdTomato (red) in all cells received intravenous injections of Alexa488-labeled bovine fetuin-A (green). Nuclei were visualized by Hoechst 33258 (blue). Videos were recorded using a two-photon microscope and a video camera. Fluorescence intensity was recorded using a GaAsP detector. **(A)** Glomerular filtration at 0, 0.4, 3, and 40 min post injection. **(B)** Time-resolved fetuin-A fluorescence in the Bowman space (BS) recorded within the white circle shown in panel **(A)**, demonstrates low level fetuin-A monomer filtration at 0.0 min increasing after 20 min. The filtered fetuin-A was reabsorbed by the proximal tubular epithelial cells (arrowheads). Still pictures illustrating CPM glomerular filtration in panel **(A)** were taken from Supplementary Movie 3.

Intravital live imaging also revealed efficient glomerular filtration and proximal tubule reabsorption of injected free fetuin-A monomer (Figure 3). Immediately post injection of free fetuin-A monomer (Figure 3, *t* = 0.4 min), green fetuin-A-associated signal was detected in glomerular capillaries. From 3 min post injection onward, the green signal accumulated in proximal tubule epithelial cells (Figure 3 white arrow, *t* = 3.0 min) continuously increasing until 40 min post injection (Figure 3 white arrows, *t* = 40.0 min). Fluorescence quantitation of filtered fetuin-A monomer in the BS revealed different filtration kinetics of free fetuin-A monomer and CPM. The fluorescence signal post CPM injection increased steadily and peaked at about 20 min reaching a mean intensity of around 70 (Figure 1B). In contrast, fluorescence following free fetuin-A monomer injection was generally lower at a mean intensity of about 30–50 and increased with a delay of about 20 min post injection, thus reaching similar levels of CPM-fluorescence at 40 min instead of 20 min post. Thus, live imaging suggested delayed filtration of free fetuin-A monomer and fast filtration of CPM. In either case, fetuin-A was re-absorbed by proximal tubule epithelial cells.

### Differential Clearance of Calciprotein Particles, Calciprotein Monomers, and Fetuin-A

**FIGURE 4:**
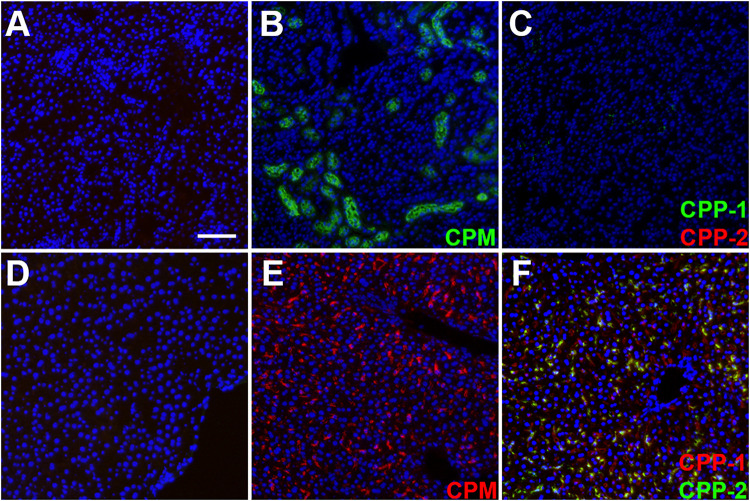
Differential clearance of free fetuin-A protein, CPM and CPP. Mice were injected with 100 μg (900 μg total protein) CPM-associated Alexa488 (green) or Alexa546-labeled fetuin-A (red). Control mice were injected with Alexa488- and Alexa546-labeled free fetuin-A monomer, or with 100 μg (total protein content) Alexa488/546-labeled CPP-1 and CPP-2. Mice were sacrificed 10 min after injection and counterstained (DAPI, blue) and kidney **(A–C)** and liver **(D,E)** frozen sections were analyzed by fluorescence microscopy. CPM were readily detected in kidney **(B)** and liver **(E)**, while CPP-1 and CPP-2 were detectable in liver **(F)** but not in kidney **(C)**. Free fetuin-A monomer was not detected in kidney or in liver **(A,D)**. Scale bar: 75 μm.

**FIGURE 5:**
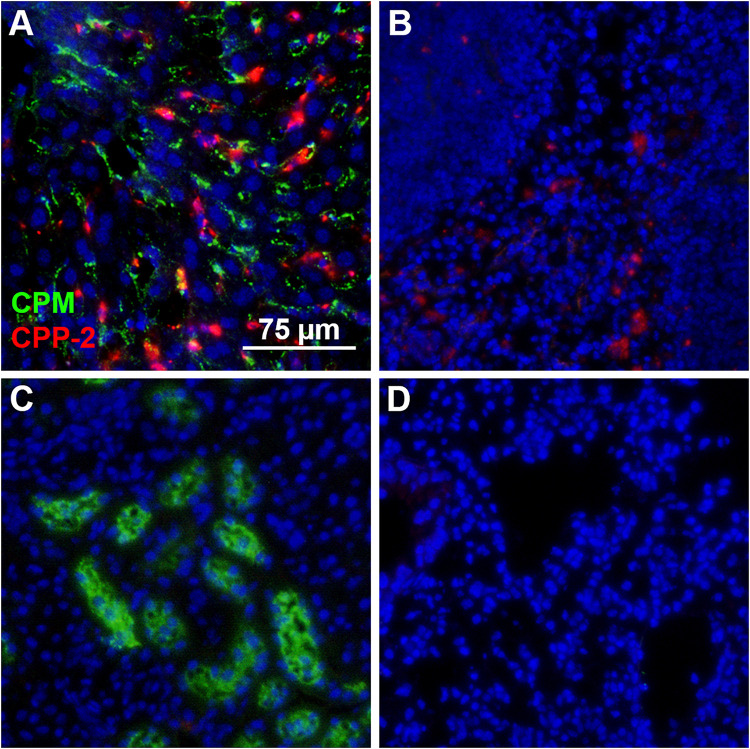
Differential clearance of CPM and CPP. Mice were simultaneously injected with CPM prepared with Alexa488-labeled fetuin-A (green) and CPP-2 prepared with Alexa546-labeled fetuin-A (red). Ten minutes post injection, liver, spleen, kidney, and lung were harvested, sectioned, counterstained with DAPI (blue) and analyzed by fluorescence microscopy. **(A)** Liver contained both CPM (green) and CPP-2 (red). **(B)** Spleen stained positive for CPP-2. CPM were highly abundant in kidney **(C)**, whereas lung **(D)** was negative for both CPM and CPP-2.

We prepared CPM by mixing Alexa488- or Alexa546-tagged (green/red) fetuin-A and injected intravenously 100 μg of CPM-bound fetuin-A (900 μg total protein) in wildtype C57BL/6 mice. To assess CPM vs. CPP clearance, we injected Alexa488/546-labeled CPP-1 and CPP-2 as controls. To compare clearance in identical animals, we injected mice with a mixture of color-coded CPM and CPP-2. Ten minutes after injection, mice were sacrificed, and organs were harvested and analyzed by frozen section fluorescence microscopy. Like CPP-1, CPM were detected in liver sinusoidal endothelial cells (Figures 4E, 5A). Unlike CPP-1 and CPP-2, however, CPM were also highly abundant in the kidney (Figures 4B, 5C), confirming rapid renal clearance established by live 2-PM (Figures 1, 2). Upon postmortem imaging, fetuin-A monomer was not detected in kidney or in liver (Figures 4A,D). CPM were absent in spleen (Figure 5B) and lung (Figure 5D) as well as in pancreas, heart and brown adipose tissue (Supplementary Figure 2).

### Inhibition of Lysosomal Proteolysis Results in Accumulation of Fetuin-A in Proximal Tubules

**FIGURE 6:**
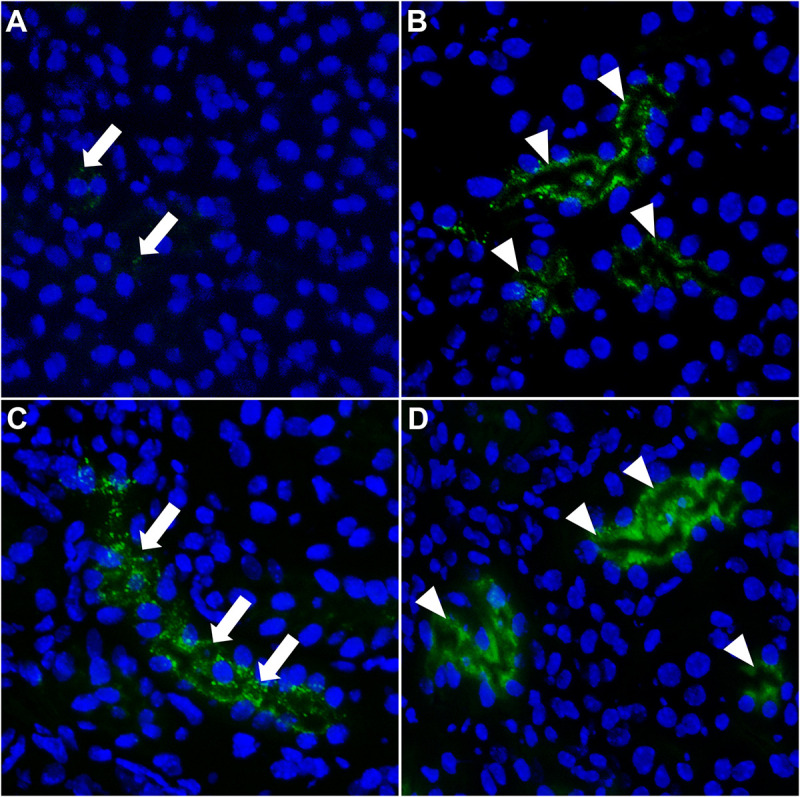
Fetuin-A accumulates in kidney tubular cells when lysosomal proteolysis is blocked. Mice were injected i.v. with saline **(A,B)** or with leupeptin **(C,D)** to inhibit lysosomal proteolysis. Thirty minutes later, mice were injected i.v. with 900 μg Alexa488-labeled fetuin-A monomer (green) or 100 μg CPM (900 μg total protein). **(A)** Saline-treated mice injected with free fetuin-A monomer showed weak and sporadic fetuin-A fluorescence in kidneys (white arrows). **(B)** Saline-treated mice injected with CPM showed strong fetuin-A fluorescence in kidney tubules (white arrow heads). When mice were pre-treated with leupeptin and lysosomal degradation was blocked, free fetuin-A monomer (white arrows) fluorescence was equally detected **(C)** like CPM-associated (white arrow heads) fetuin-A fluorescence **(D)**.

To clarify perceived differences in live and *ex vivo* imaging of CPM and free fetuin-A monomer clearance kinetics, we injected mice with saline as vehicle control or with 0.03 mg/g bodyweight leupeptin to inhibit lysosomal proteolysis. Thirty minutes post injection, mice were injected with 900 μg Alexa488-tagged free fetuin-A monomer or with 100 μg CPM (900 μg total protein). After 20 min, mice were sacrificed, and frozen kidney sections were imaged for fetuin-A-associated green fluorescence. Injection of free fetuin-A monomer (Figure 6A) in saline-treated mice resulted in low and sporadic fetuin-A-associated green signal (white arrows in Figure 6A). In contrast, a bright green signal was always detected in kidneys of CPM injected mice (white arrow heads in Figure 6B), corroborating previous observations in *ex vivo* imaging (Figure 4). When mice were pre-treated with leupeptin inhibiting lysosomal proteolysis, free fetuin-A monomer (white arrows in Figure 6C) and CPM-derived fetuin-A (white arrow heads in Figure 6D) were both readily detected in kidney sections. Collectively, these results account for the perceived differences in fetuin-A detection between live- and *ex vivo* imaging in that a small amount of free fetuin-A monomer is continuously filtered by the kidney and is quickly re-absorbed and cleaved by proteolysis in proximal tubule epithelial cells. CPM is more readily cleared than free fetuin-A, which is spuriously detected in untreated mice at 10 min post injection, but readily detected after leupeptin inhibition of lysosomal proteolysis in proximal kidney tubular cells at 20 min post injection. Our results thus suggest that little free fetuin-A monomer, but all CPM pass the glomerular filtration barrier.

### Calcium Phosphate Binding Neutralizes Negative Charge of Fetuin-A

The clearance studies in live mice demonstrated glomerular filtration and re-absorption in the tubular system of CPM-associated fetuin-A. In contrast, less injected free fetuin-A monomer passed into kidney tubular cells most likely due to charge repulsion at the glomerular filtration membrane of negatively charged free fetuin-A monomer (Heiss et al., 2003). Anion exchange chromatography confirmed the negative charge of fetuin-A. Free fetuin-A monomer (900 μg) bound the positively charged column material as indicated by the missing UV280 (protein measurement) peak during sample application and column washing steps (Supplementary Figure 3A, blue line). Column elution with a salt step gradient (orange conductivity line in Supplementary Figure 3A) recovered 98% of the applied fetuin-A protein in one single peak (Supplementary Figure 3, blue line), demonstrating elution of the negative fetuin-A protein from the positive ion exchange column. In contrast, 10-15% of the loaded protein eluted with the flow through fraction when we loaded 100 μg (900 μg total protein) CPM-bound fetuin-A to the ion exchange column (Supplementary Figure 3B, blue line) demonstrating that CPM did not interact with the positively charged column material. Column elution with a salt step (orange conductivity line in Supplementary Figure 3B) recovered the remaining 85–90% of loaded protein, which represented negatively charged free fetuin-A monomer also present in the CPM preparation. These results suggest that the overall negative charge of fetuin-A was neutralized by bound calcium phosphate, allowing CPM to pass the renal filtration barrier. CPM samples were analyzed for protein-, calcium-, and phosphate contents before and after ion exchange chromatography (Table 1). Protein measurements confirmed chromatography results, detecting about 15% protein in the CPM fraction (Table 1). The CPM fraction (first blue peak in Supplementary Figure 3B) also contained about 45% of the total calcium and about 40% of the total phosphate suggesting strong calcium phosphate binding by fetuin-A. The free fetuin-A fraction (second blue peak in Supplementary Figure 3B) contained about 12% of the total applied calcium and about 20% of the applied phosphate. These latter measurements were close to the detection limit of the assays (protein assay: 0.065 mg/ml; calcium assay: 0.193 mM; phosphate assay: 0.274 mM) and may in fact represent background level.

**TABLE 1:** Analysis of ion exchange chromatography starting solutions and fractions.

	pre ÄKTA	post ÄKTA (CPM fraction)	post ÄKTA (Fetuin-A fraction)
Fetuin-A (mg/ml)	0.622	0.091 (15.8%)	0.463 (74.6%)
Ca (mM)	1.463	0.671 (45.9%)	0.178 (12.2%)
P (mM)	0.786	0.302 (38.5%)	0.156 (19.8%)

### Calciprotein Monomers Are Endocytosed by Cultured Proximal Tubular Epithelial Cells

**FIGURE 7:**
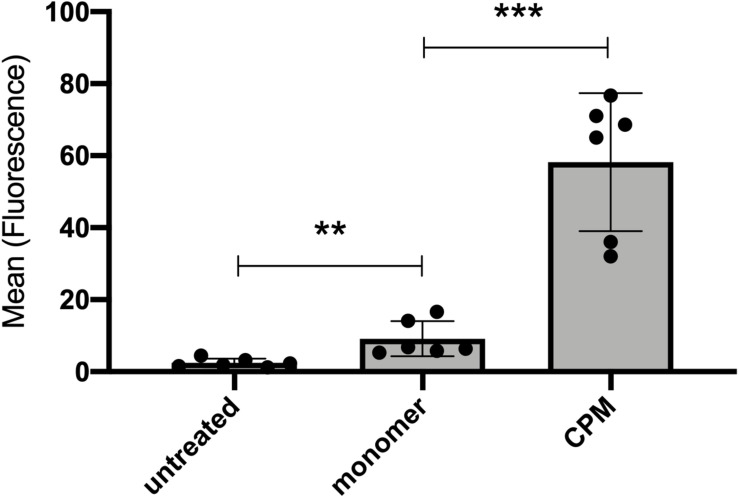
Cultured kidney epithelial cells endocytose free fetuin-A monomer and CPM-associated fetuin-A. Proximal tubular kidney epithelial (HK-2) cells were incubated with 100 μg (900 μg total protein) CPM prepared with Alexa488-tagged fetuin-A or free fetuin-A monomer and cellular uptake was analyzed by flow cytometry. HK-2 cells endocytosed significant amounts of free fetuin-A monomer, and even more efficient CPM-associated fetuin-A. Box plots illustrate six independent experiments, mean ± standard deviation. **p < 0.01, ***p < 0.001, analyzed with unpaired *t*-test in GraphPad Prism v9.

Experiments with live mice demonstrated a rapid and efficient uptake of CPM-derived fetuin-A by proximal tubular epithelial cells. To verify this observation in isolated cells, we treated HK-2 human kidney proximal tubular cells with cell culture medium (untreated), 100 μg (900 μg total protein) Alexa488-tagged CPM-fetuin-A and free fetuin-A monomer, and studied cellular uptake by flow cytometry. Figure 7 demonstrates that HK-2 cells endocytosed small amounts of free fetuin-A monomer, and at least 6-times more CPM-associated fetuin-A.

Figures 4, 5 suggested concurrent uptake of CPM and CPP-1 in the liver by LSEC. To address this in isolated cells, we treated CPP-endocytosing macrophages, LSEC, and HK-2 cells with the same protein–mineral complexes mentioned above and measured cellular uptake by flow cytometry. Supplementary Figure 4 demonstrates that HK-2 cells also endocytosed CPP-1 and CPP-2, even more efficiently than free fetuin-A monomer and CPM. Similarly, LSEC (Supplementary Figure 4B) and macrophages (Supplementary Figure 4C) endocytosed small amounts of free fetuin-A monomer, roughly twice as much CPM, and 8-fold and 10-fold more particulate CPP-1 and CPP-2, respectively, confirming inverse differential preference of macrophages (Koeppert et al., 2018). These results suggest that all investigated cell types in principle are able to endocytose non-particulate fetuin-A monomer and CPM as well as particulate CPP-1 and CPP-2 corroborating results of live mouse clearance experiments. The only striking discrepancy detected in live vs. cell culture clearance pertains to CPP clearance in the kidney, which was never observed in live mice, but readily detected in HK-2 cell culture. This discrepancy is due the fact that large particular CPP will never pass the renal filtration barrier and will therefor never contact tubular epithelial cells in live animals.

### Fetuin-A Protects From Calcium Phosphate-Induced Cell Damage

**FIGURE 8:**
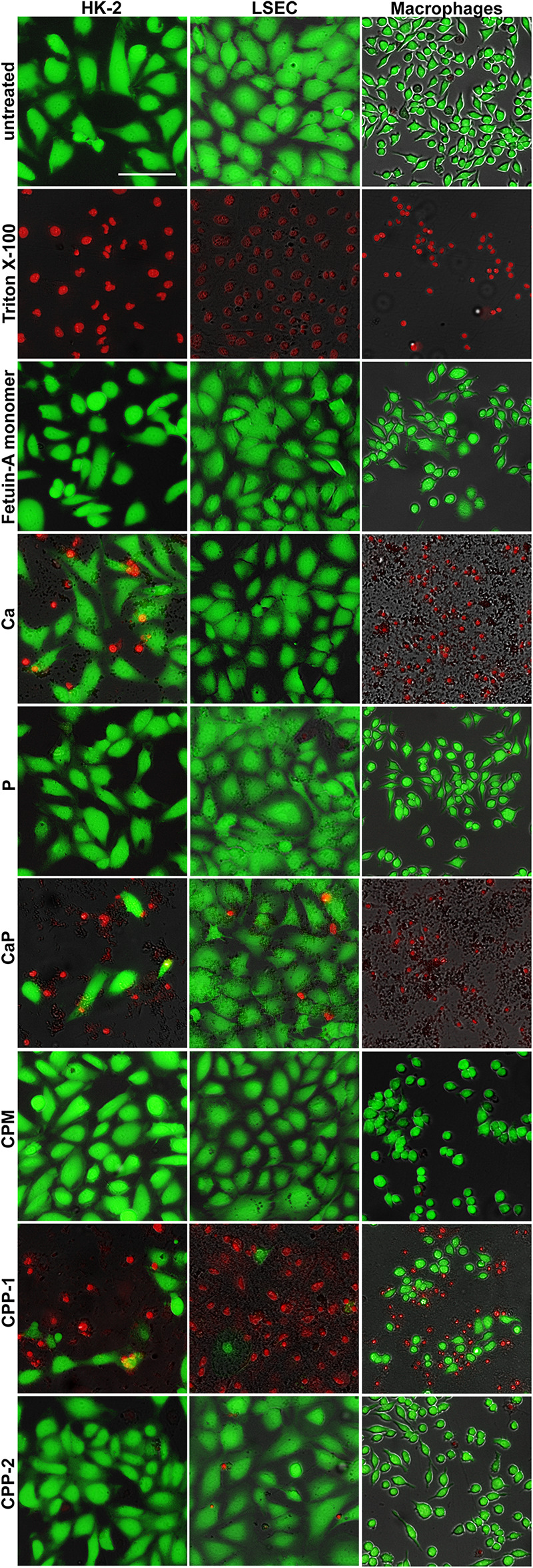
CPM formation attenuates calcium phosphate-induced cytotoxicity. Cells involved in CPP and CPM clearance (HK-2 cells, LSEC, macrophages), were left untreated to assess maximum FDA staining, or were treated with Triton X-100 for 1 min for maximum PI staining. Cells were exposed for 8 h to calcium content matched (2.5 mM) CPM, CPP-1, CPP-2, and controls [free fetuin-A monomer, 2.5 mM calcium, 2.0 mM phosphate, and a combination of calcium and phosphate (CaP)]. Calcium was strongly toxic in macrophages, moderately toxic in HK-2 cells, and non-toxic in LSEC. CaP was strongly toxic in macrophages and moderately toxic in HK-2 cells and LSEC. An identical amount of calcium (or CaP) applied as CPM, a protein-mineral complex stabilized by fetuin-A was universally non-toxic despite cellular uptake. CPP-1 were highly toxic in all cells, whereas CPP-2 were not toxic. Scale bar: 100 μm.

We analyzed in cell culture of cells involved in CPM and CPP clearance the cytotoxicity of CPM, CPP, and their components (Figure 8). To this end, macrophages, LSEC, and HK-2 cells were treated with calcium content-matched (2.5 mM) CPM, CPP-1, CPP-2, and the corresponding controls (2.5 mM Ca, 2.0 mM P, CaP, free fetuin-A monomer) over a period of 8 h. FDA/PI staining revealed strikingly different cytotoxicity in the various cell types, especially after treatment with calcium and calcium-phosphate (Figure 8). HK-2 cells suffered less toxicity following calcium (Ca) and calcium-phosphate (CaP) exposure than did macrophages, which all died following treatment with calcium or calcium-phosphate. Calcium also did not harm LSEC, and calcium-phosphate induced rather mild toxicity in LSEC compared to macrophages and HK-2 cells. CPM, on the other hand, led to similar results in all cell types in that they never induced any toxicity despite carefully matched CPM-calcium and phosphate concentrations. Collectively, these results corroborate the role of fetuin-A as a mineral chaperone stabilizing calcium phosphate as a colloid, more specifically forming CPM thereby permitting conditions of supersaturation while preventing cell damage. Free fetuin-A monomer did not induce any toxicity in any of the studied cell types. Treatment with CPP-1 was highly toxic in all cell types, whereas CPP-2 were not toxic as reported previously (Koeppert et al., 2018).

### Calciprotein Monomers Do Not Activate the NLRP3 Inflammasome

**FIGURE 9:**
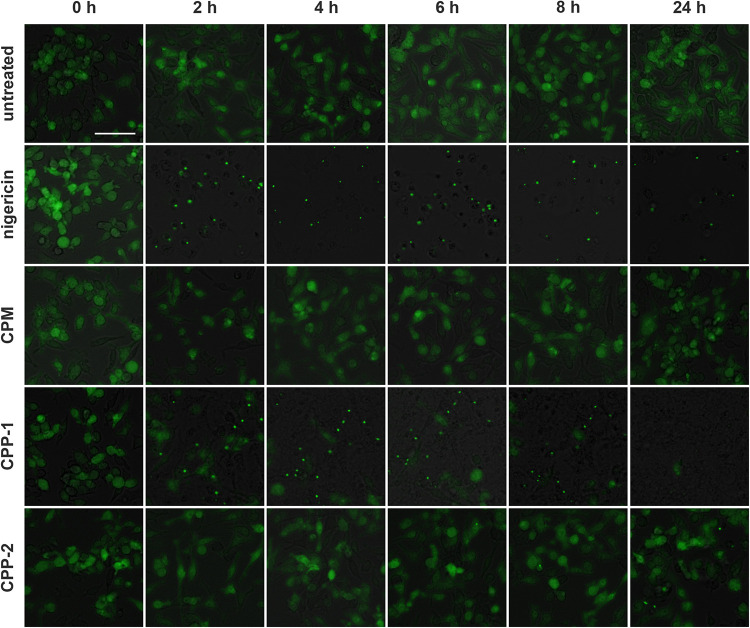
Calciprotein monomer does not activate the NLRP3 inflammasome. Immortalized macrophages expressing the inflammasome adaptor protein labeled *apoptosis-associated speck like protein containing a caspase recruitment domain* ASC fused to green fluorescent protein, were exposed to medium (untreated), 10 μM nigericin, or to calcium content matched (2.5 mM) CPM, CPP-1, or CPP-2 and were monitored over a time period of 24 h. Single fluorescent speck formation suggested that CPP-1 triggered fast inflammasome assembly starting at about 2 h after particle addition, and comparable to nigericin positive control treatment, which caused immediate influx of Ca and hence rapid inflammasome assembly. CPP-2 showed a mild and delayed inflammasome assembly starting at about 8 h. In contrast, exposure to CPM did not result in inflammasome assembly. Scale bar: 50 μm.

We previously reported that CPP-1 predominantly induced NLRP3 inflammasome assembly and subsequent cytokine secretion, while CPP-2 predominantly stimulated immediate secretion of preformed TNFα (Koeppert et al., 2018). To assess the inflammatory potency of CPM, we studied immortalized ASC-GFP macrophages. In the unstimulated stage, these cells show a diffuse green fluorescent signal throughout the cytoplasm. Following pro-inflammatory stimulation, the cells assemble their inflammasome, presenting as one single brightly fluorescent (green) spot per cell. We treated ASC-GFP macrophages with calcium content-matched (2.5 mM) CPM, CPP-1, and CPP-2 and monitored the cells for up to 24 h (Figure 9). CPP-1 rapidly triggered inflammasome assembly within 2 h, while CPP-2 triggered delayed inflammasome assembly after 8 h confirming published results (Koeppert et al., 2018). In contrast, CPM containing identical 2.5 mM calcium and 2 mM phosphate (Figure 9, third row from top) did not trigger assembly of the inflammasome at any time during the observation period, suggesting high stability of the fetuin-A/calcium-phosphate complex and concomitant low inflammatory potential of CPM compared to Ca, CaP, or CPP-1.

## Discussion

Protein-mineral complexes are natural byproducts of mineral homeostasis. In analogy to lipoprotein particles, we named fetuin-A containing calciprotein monomer CPM, and calciprotein particles CPP (Jahnen-Dechent et al., 2011). Local deposition of CPP is usually a consequence of prolonged calcium and phosphate supersaturation in the body leading to ectopic calcification. Supplementary Figure 5 illustrates the clearance and metabolism of calciprotein particles CPM and CPP *in vivo*. Fetuin-A deficiency and thus the lack of stable CPM and CPP likewise results in ectopic calcification (Schäfer et al., 2003). Accordingly, a CPM/CPP clearance mechanism is required mediating removal from the circulation to prevent deposition and buildup. Here we studied for the first time the systemic and cellular clearance of synthetic CPM, their inflammatory potential, and their cytotoxicity. We previously reported rapid blood clearance and contribution to calcification of established atherosclerotic plaque of CPP-2 (Herrmann et al., 2012). Ongoing research unravels the relevance of distinct forms of CPM and CPP in physiology and disease. CPP-1 and even earlier forms termed low density CPP or CPM, are associated with CKD in patients who have an increased risk of calcification (Miura et al., 2018). We and others suggested that CPM may be the predominant circulating form of physiological protein-mineral complexes in the body. The presence of primary and especially CPP-2 forms may signify progressively more pathological stages of mineral metabolism up to lethal calcifying sclerosing peritonitis (Olde Loohuis et al., 2010; Miura et al., 2018; Smith et al., 2018). CPM are much smaller (about 9 nm) and softer than both CPP-1 (50–100 nm) and CPP-2 (120–150 nm) (Jahnen-Dechent et al., 2020), and contain only fetuin-A unlike CPP, which contain complex plasma protein mixtures including fetuin-A. Thus, CPM and CPP may be differentially cleared based on these characteristics alone. Here, we demonstrated renal CPM filtration. Immediately post injection, CPM and, with different clearance kinetics, also free fetuin-A monomer accumulated in kidney tubules, whereas CPP never passed into kidney tubules. Large CPP size likely prevented renal filtration while charge neutralization by mineral loading facilitated CPM clearance compared to free fetuin-A clearance. It is tempting to speculate that free fetuin-A monomer may in fact not be filtered at all due to charge repulsion, and that the fluorescence detected with delayed kinetics during live clearance experiments in truth represented CPM that assembled in the mice immediately following injection of labeled free fetuin-A monomer. This point merits further study.

Clearance of CPM and fetuin-A both suggested re-uptake of fetuin-A by kidney proximal tubule epithelial cells. This was most likely mediated by the well-established megalin pathway as shown before in rodents (Matsui et al., 2013). In this study, Matsui and colleagues detected fetuin-A in proximal tubule epithelial cells by immunofluorescent staining, which decreased when endocytosis through megalin was blocked. Like here, injected free fetuin-A monomer was only detected when lysosomal proteolysis was blocked and epithelial cells accumulated fetuin-A, supporting our conclusion that glomerular free fetuin-A monomer filtration occurs below the detection limit. We conclude that CPM are taken up earlier and at a higher rate and therefor are better visible even without leupeptin treatment. This is in full agreement with results described in Matsui et al. (2013), Mizuno et al. (1991) and Rudloff et al. (2021) that intriguingly show increased fetuin-A staining intensity in proximal tubule lumen if re-uptake is inhibited by His-Rap megalin (Matsui et al., 2013) or by Clcn5 knockout (Ewence et al., 2008), or is increased in proximal tubule epithelial cells, if lysosomal degradation is inhibited by leupeptin (Matsui et al., 2013). Nevertheless, small amounts of free fetuin-A, like albumin [around 3 μg/ml in proximal tubular fluid in rats (Leber and Marsh, 1970)], may pass the renal filtration barrier, and will also be re-absorbed by proximal tubule epithelial cells. For reasons outlined above, fetuin-A detected in those cells is likely CPM-derived for the most part. We hypothesize that CPM readily pass into the glomerular filtrate, because mineral binding confers to fetuin-A an overall neutral charge and thus prevents charge repulsion in the glomerular filtration barrier. After transfer into the primary urine, CPM likely dissociate due to pH or ion activity driven solubility changes. Fetuin-A will be re-absorbed by tubular epithelial cells, and excess mineral will be excreted in urine. Alternatively, CPM are endocytosed as a whole and dissociated in the lysosome. Both mechanism is fully compatible with the role of fetuin-A as a mineral chaperone that helps stabilization, transport and elimination of excess calcium phosphate mineral. It also explains the detection of fetuin-A protein in kidney tubules in localization studies (Mizuno et al., 1991), despite the fact that fetuin-A is not made in the kidney.

We and others demonstrated the inflammatory potential of CPP-1 and CPP-2 (Smith et al., 2013; Koeppert et al., 2018). The incorporation and lysosomal degradation of uncoated calcium phosphate crystals triggered intracellular calcium transients and induced secretion of inflammatory cytokines including TNFα, IL1β, and IL8 (Nadra et al., 2005; Pazar et al., 2011). The increase in intracellular calcium following exposure to calcium phosphate crystals induced cell death in vascular smooth muscle cells (Ewence et al., 2008). Here, we demonstrated that unlike CPP, CPM are neither inflammatory nor cytotoxic suggesting a benign physiological salvage pathway of excess calcium phosphate by CPM. It has been shown that fetuin-A-binding of calcium phosphate can even reduce the high cytotoxicity and inflammatory potential of preformed calcium phosphate crystals (Dautova et al., 2014).

Another major finding was, that all cell types associated with CPM or CPP clearance (macrophages, LSEC, kidney epithelial cells) endocytosed both CPM and CPP when directly exposed in cell cultures. However, *in vivo* CPP injection experiments never detected CPP in kidneys, hence proximal tubule epithelial cells *in vivo* will never contact CPP unless they are formed *in situ*, which has in fact been observed in kidney damage models (Rudloff et al., 2021). In contrast LSEC, or endothelial cells in general, and macrophages are certainly among the first cells contacting CPM and CPP. The uptake of CPP from circulation by these cell types is well known (Herrmann et al., 2012; Koeppert et al., 2018). We suggest that endothelial cells and macrophages will readily clear CPP from circulation, should they occur either due to insufficient or delayed renal CPM clearance. Endocytosis of small amounts of CPP may be non-toxic, yet may cause delayed recycling of excess mineral into the circulation with subsequent renal filtration.

The demonstration of renal CPM clearance, the transient presence of fetuin-A in primary urine and subsequent co-localization of fetuin-A with kidney tubular epithelial cells observed here is strong evidence for a physiological salvage mechanism and renal clearance of the mineral component of CPM, and subsequent recovery of free fetuin-A by reabsorption. This mechanism requires experimental verification on several accounts. In any case, we present a viable and elegant elimination mechanism for excess calcium phosphate mineral, which may form at any time due to the low solubility of calcium phosphate in circulation (Jahnen-Dechent et al., 2020), in the extracellular space particularly following cell and tissue damage (Ghadially, 2001), following bouts of hyperphosphatemia, e.g., post-prandially (Smith et al., 2018), and especially in chronic kidney disease (Gatate et al., 2020).

## Data Availability Statement

The raw data supporting the conclusions of this article will be made available by the authors, without undue reservation.

## Ethics Statement

The animal study was reviewed and approved by by the Landesamt für Natur-, Umwelt- und Verbraucherschutz (LANUV, 84-02.04.2013.A113 and 84.02.04.2015.A294).

## Author Contributions

SK performed the experiments, analyzed the data, and drafted and revised the manuscript. AG performed the experiments, analyzed the data, and revised the manuscript. SP, CW, AB, and SG performed the experiments. JH designed the study and revised the manuscript. WJ-D designed the study, analyzed the data, drafted and revised the manuscript. All authors contributed to the article and approved the submitted version.

## Conflict of Interest

The authors declare that the research was conducted in the absence of any commercial or financial relationships that could be construed as a potential conflict of interest.
