# Supplementary figures and images for: Live Imaging of Calciprotein Particle Clearance and Receptor Mediated Uptake: Role of Calciprotein Monomers
Source: Front Cell Dev Biol. 2021 Apr 29;9:633925. doi: 10.3389/fcell.2021.633925 (PMC8116800; doi:10.3389/fcell.2021.633925)

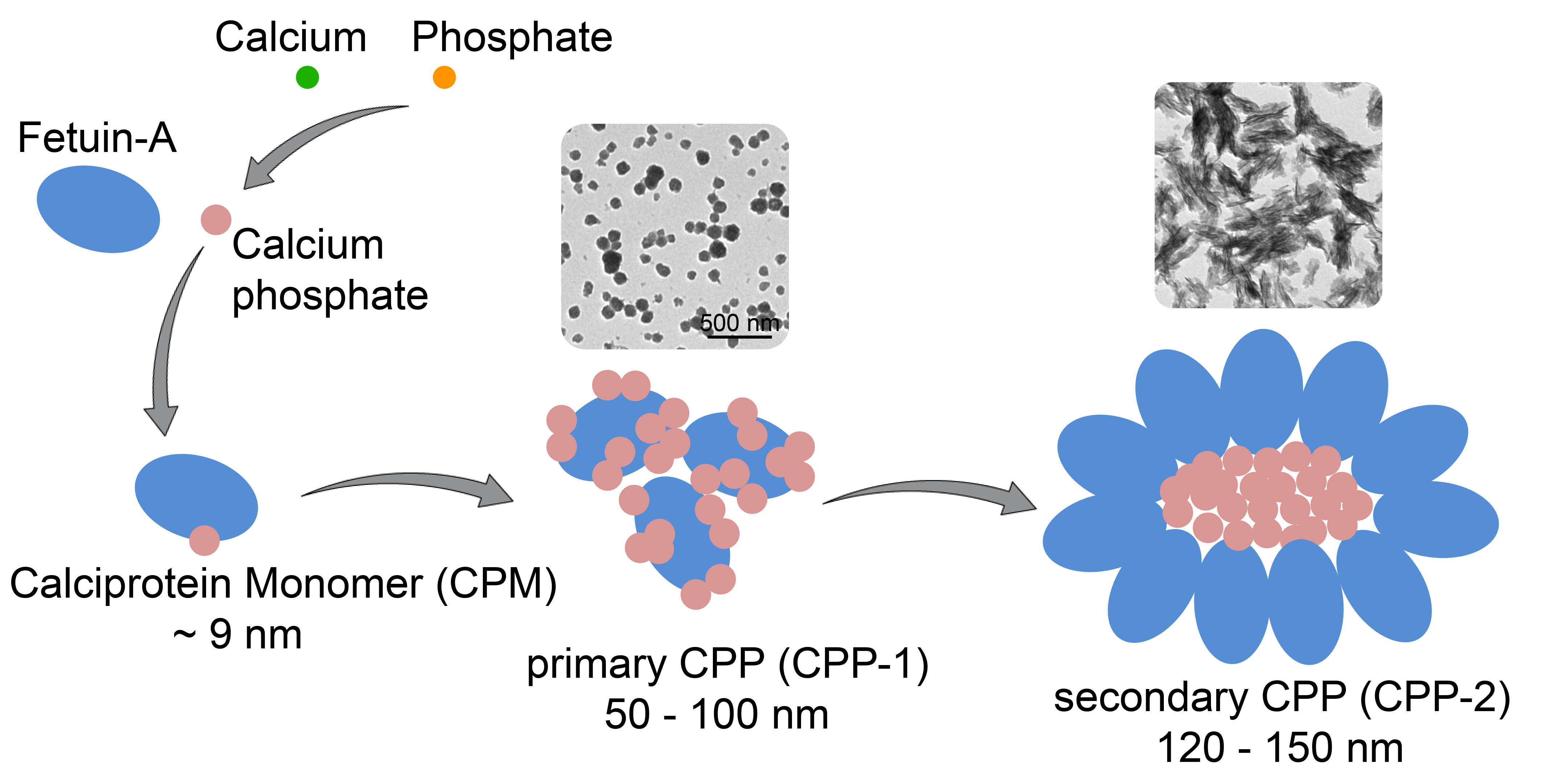

Supplement: Supplementary file 1 [file Image_1.TIF]

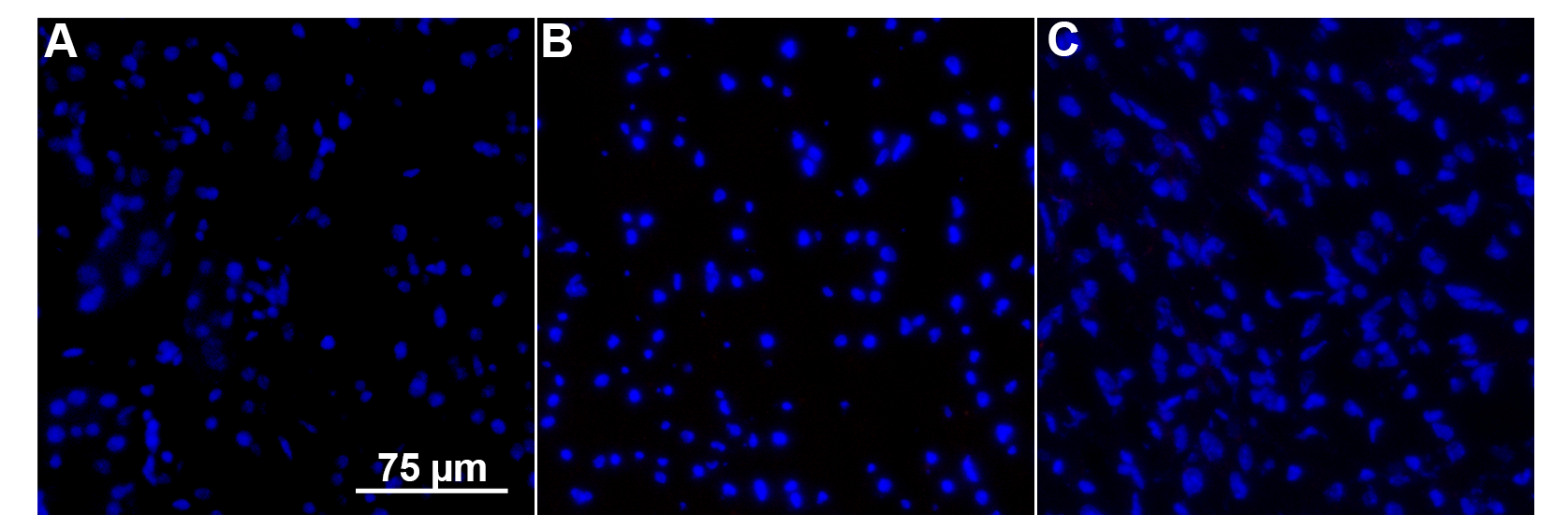

Supplement: Supplementary file 2 [file Image_2.TIF]

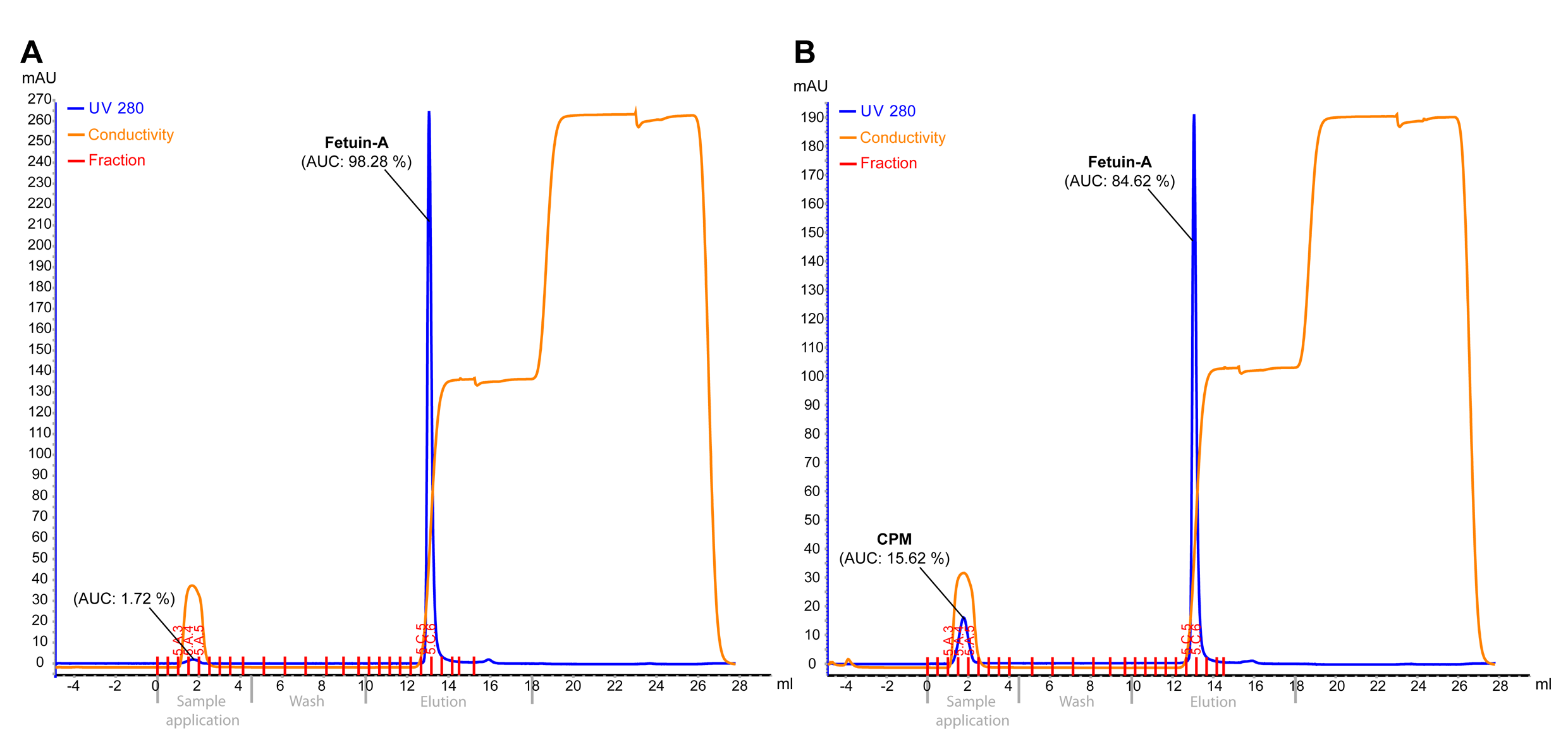

Supplement: Supplementary file 3 [file Image_3.TIF]

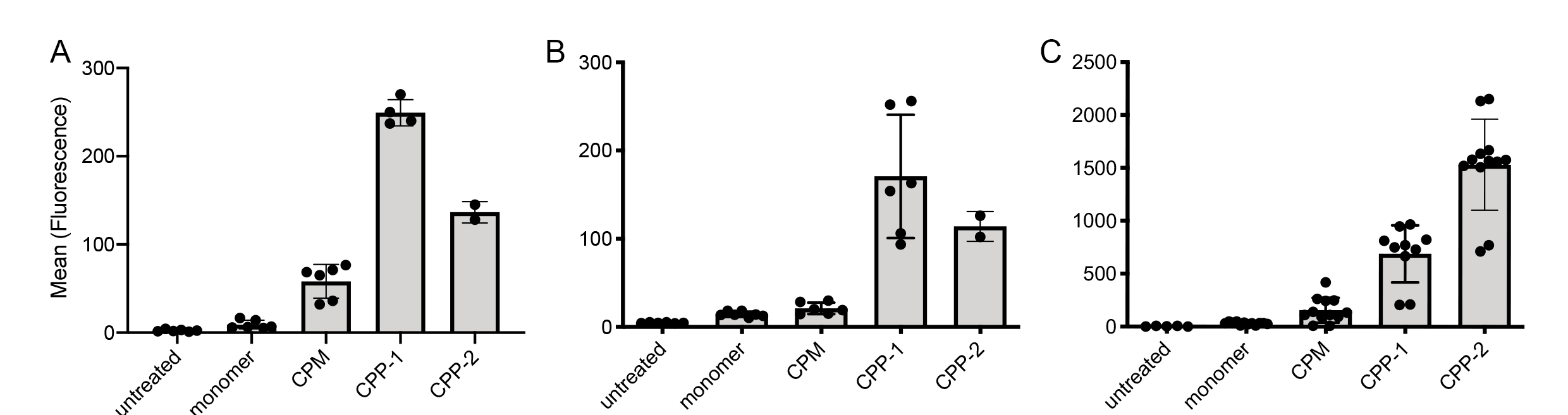

Supplement: Supplementary file 4 [file Image_4.TIF]

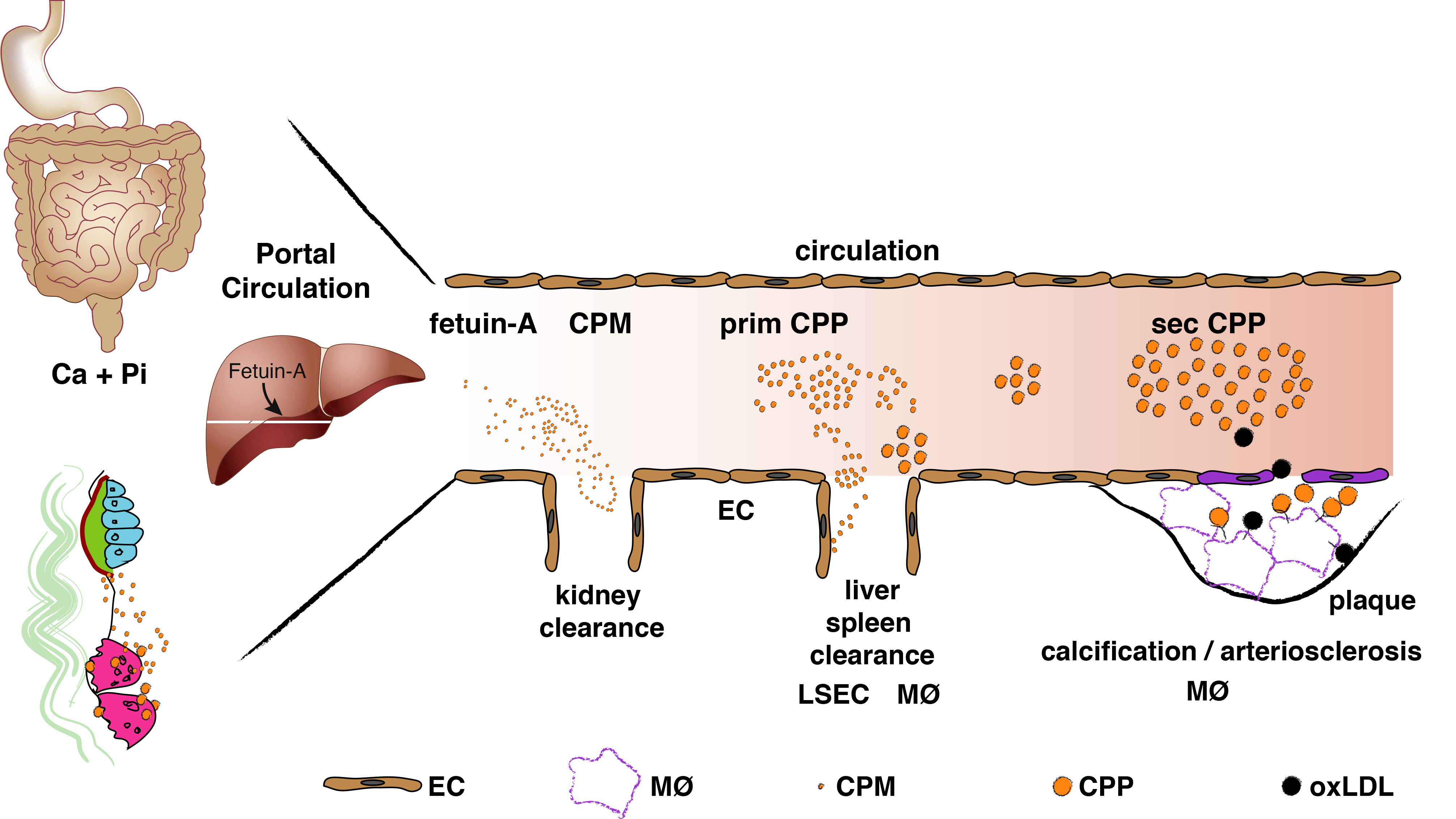

Supplement: Supplementary file 5 [file Image_5.TIF]
