# Supplementary material for: Live Imaging of Calciprotein Particle Clearance and Receptor Mediated Uptake: Role of Calciprotein Monomers
Source: Front Cell Dev Biol. 2021 Apr 29;9:633925. doi: 10.3389/fcell.2021.633925 (PMC8116800; doi:10.3389/fcell.2021.633925)
Supplement: Supplementary file 6 [file Table_1.DOCX]

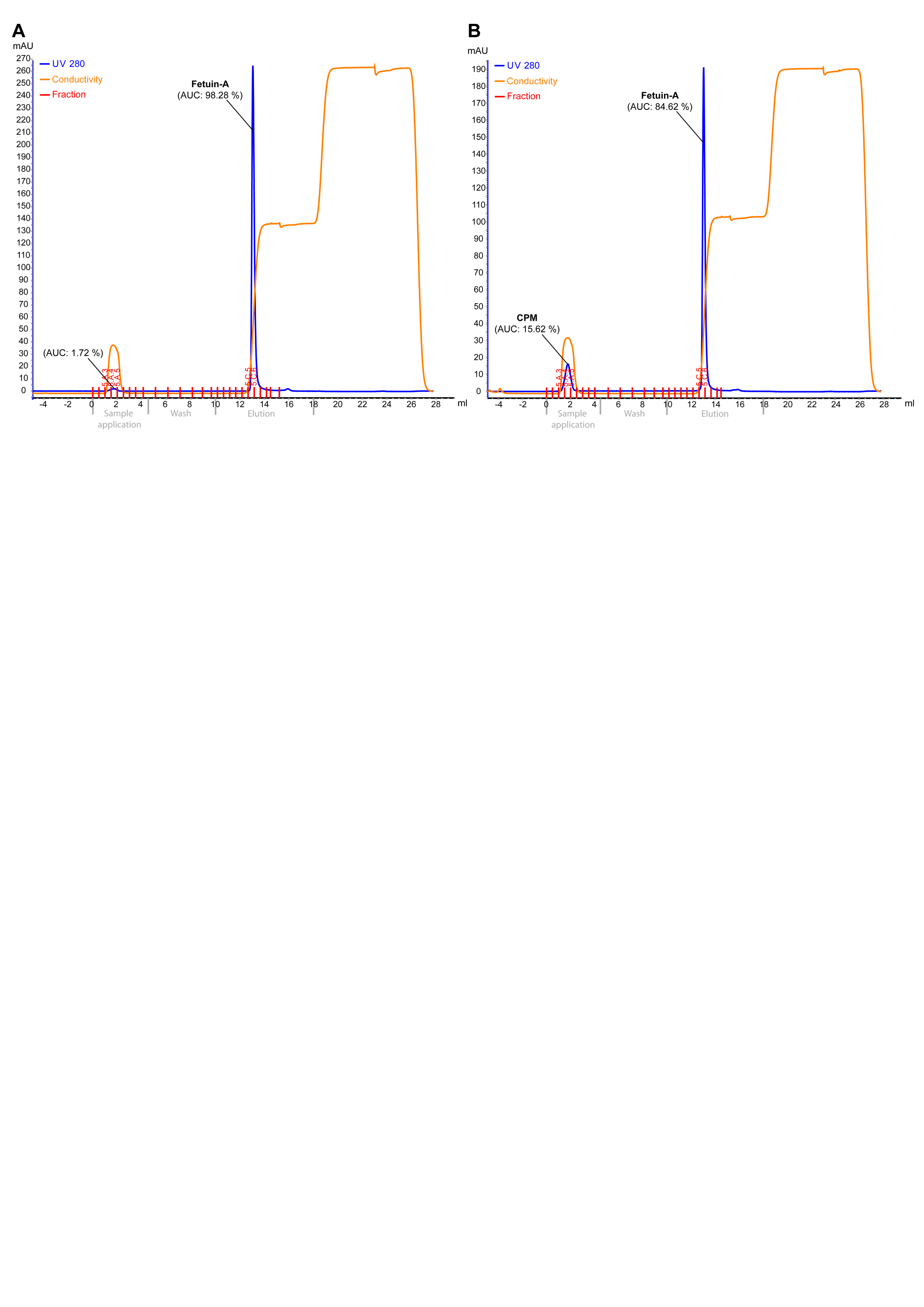


**SFigure 1. Anion exchange chromatography of fetuin-A and CPM.** A**)** Free fetuin-A monomer (900 µg), or B) 100 µg (900 µg total protein) CPM-bound fetuin-A were applied to an anion exchange column. A) Free fetuin-A monomer showed a strong binding to the positively charged column material indicated by the negligible UV280 peak during sample application and column wash steps (blue line). During elution (increasing orange conductivity line), a peak containing 98 % of applied fetuin-A appeared (blue line), demonstrating that negative fetuin-A protein was entirely bound to positive column material. B) When applying 100 µg (900 µg total protein) CPM-bound fetuin-A to the column, a protein peak (blue line) was detectable during the sample application step containing 15 % of the loaded protein amount and showing CPM to not interact with the positively charged column material. In the following elution step (increasing orange conductivity line), the remaining free fetuin-A monomer (85 % of loaded protein) was washed from the column.


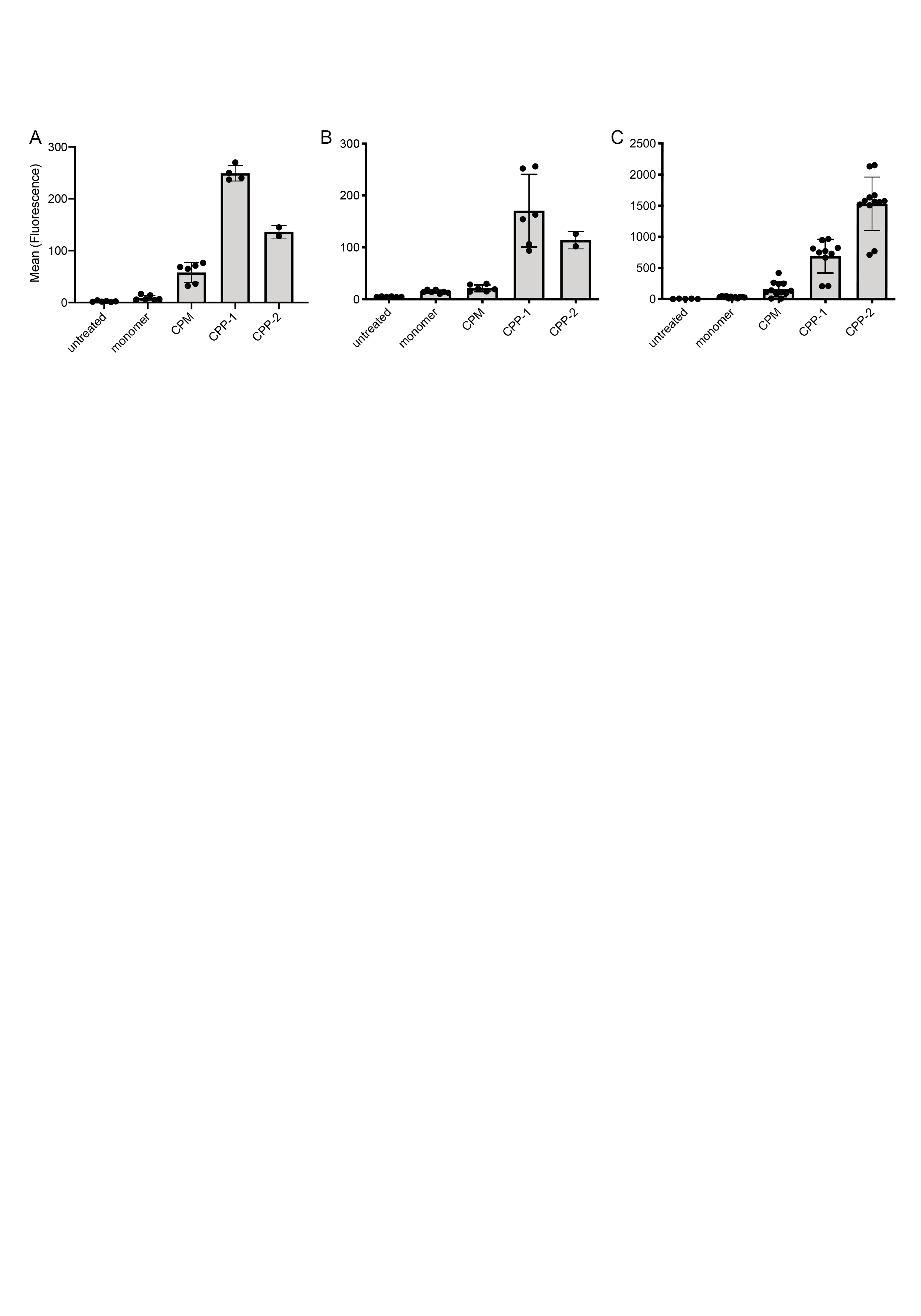


**SFigure 2. Cultured kidney epithelial cells, liver sinusoidal endothelial cells and bone marrow-derived macrophages endocytose CPM and CPP.** Proximal tubular kidney epithelial cells (**A**), liver sinusoidal endothelial cells (**B**), and bone marrow-derived macrophages (**C**) were incubated with 100 µg (900 µg total protein) CPM prepared with Alexa488-tagged fetuin-A, free fetuin-A monomer, CPP-1, and CPP-2 and cellular uptake was analyzed by flow cytometry. HK-2 cells endocytosed significant amounts of free fetuin-A monomer and CPM-associated fetuin-A (Figure 5). Additionally, HK-2 cells also endocytosed even higher amounts of both, CPP-1 and CPP-2 (**A**), however, in vivo kidney epithelial cells will not get in contact with CPP as demonstrated by in vivo particle injection experiments, where CPP could be never detected in kidneys. Besides efficient CPP endocytosis by LSEC (**B**) and macrophages (**C**) as published before (7, 8), both cell types also endocytosed CPM-associated fetuin-A, consistent with in vivo injection experiments showing also a positive CPM signal in liver cells.
